# Skin ECM Provides a Bio-Derived Platform for Supporting Dermal Renewal and Matrix Synthesis

**DOI:** 10.4014/jmb.2505.05015

**Published:** 2025-06-17

**Authors:** Sewon Park, Mi Jeong Lee, Ha Jin Kim, Soojeong Choi, Jung Ho Cho, Seungrok Lee, Seulbi Lee, Yoonhee Jin, Seung-Woo Cho

**Affiliations:** 1 Department of Biotechnology, Yonsei University, Seoul 03722, Republic of Korea; 2 Department of Integrated Biotechnology, Yonsei University, Incheon 21983, Republic of Korea; 3 Cellartgen Inc., Seoul 03722, Republic of Korea; 4 Department of Physiology, Graduate School of Medical Science, Brain Korea 21 Project, Yonsei University College of Medicine, Seoul 03722, Republic of Korea; 5 Center for Nanomedicine, Institute for Basic Science (IBS), Seoul 03722, Republic of Korea

**Keywords:** Skin extracellular matrix, dermal fibroblast, skin regeneration, wound healing

## Abstract

The extracellular matrix (ECM) plays a central role in directing dermal fibroblast behavior and coordinating tissue regeneration through its structural organization and biochemical signaling. In this study, we investigate the composition and regenerative bioactivity of Skin ECM in the context of dermal remodeling as a soluble supplement and surface coating for fibroblast culture. Proteomic profiling demonstrates that Skin ECM preserves the molecular complexity and skin-specific composition of native dermal ECM, including key structural and signaling proteins essential for tissue repair, with over 95% overlap with the human skin proteome, highlighting its strong tissue specificity and biological relevance. Functionally, Skin ECM enhances fibroblast migration during wound healing, upregulates elastin expression, and suppresses transforming growth factor beta 1 (TGF-β1)–induced expression of profibrotic and inflammatory markers, indicating inhibition of fibroblast activation. *In vivo* subcutaneous implantation confirms high local and systemic biocompatibility without signs of inflammation or toxicity. Collectively, these findings establish Skin ECM as a bioactive, tissue-specific, and immunologically compatible ECM material, offering broad utility in regenerative medicine, three-dimensional (3D) skin model systems, and dermal therapeutics including cosmetic interventions.

## Introduction

The extracellular matrix (ECM) is a structurally and functionally dynamic three-dimensional (3D) network composed of fibrous proteins, glycoproteins, and proteoglycans that provides mechanical stability, tensile strength, and biochemical cues to surrounding cells [1, 2]. In skin, dermal fibroblasts are the principal producers of ECM components, including collagens, elastin, fibronectin, and glycosaminoglycans, which together form a highly organized environment that maintains tissue architecture and regulates cell behavior [3-5]. Beyond its mechanical function, the ECM plays a pivotal regulatory role by modulating key cellular processes such as adhesion, migration, differentiation, and collagen synthesis through integrin-mediated signaling and growth factor sequestration [6-8].

To recapitulate the complex microenvironment of native tissues *in vitro*, decellularization has emerged as an effective strategy for generating biologically active scaffolds. By removing cellular content while preserving ECM composition, decellularized matrices provide a tissue-specific niche enriched with structural proteins, bioactive ligands, and growth factor-binding domains [9-11]. Decellularized skin ECM, in particular, retains the intricate fibrillar network and biochemical diversity of the dermis, including abundant collagen types I, III, and VII, elastin, and matrix-bound regulators of cell function. Previous reports have shown that hydrogels derived from decellularized human dermis recapitulate not only the structural framework but also the molecular signature of native skin [12, 13]. In this preserved ECM milieu, integrin ligands, matricellular proteins, and immobilized growth factors remain bioavailable to guide cell behavior, making decellularized skin ECM an effective mimic of the native dermal microenvironment.

Decellularized dermal ECM has been explored in various forms, including grafts, hydrogels, and composite scaffolds for promoting skin regeneration [14-16]. These studies highlight its potential as a biologically instructive matrix capable of modulating cell behavior and facilitating wound healing. However, despite increasing interest in dermal ECM-based biomaterials, most applications have focused on 3D or implantable formats, with relatively limited emphasis on its use as a soluble supplement or surface coating for *in vitro* fibroblast culture. Yet, these alternative formats offer distinct advantages for research and translational use, particularly in enabling versatile and high-throughput screening platforms.

In this study, we explored Skin ECM, a decellularized dermal matrix formulated as a soluble supplement and surface coating for fibroblast culture. Proteomic analysis of Skin ECM revealed the retention of key structural and signaling proteins associated with skin regeneration, including fibrillar collagens, elastin, lumican, fibronectin, and laminin. Functionally, Skin ECM significantly enhanced fibroblast migration in *in vitro* wound healing assays and increased type I collagen production compared to recombinant human collagen, highlighting its ability to provide biologically instructive cues beyond those of single-component substrates. Moreover, *in vitro* cytocompatibility and *in vivo* implantation studies confirmed that Skin ECM supports cell viability and elicits no adverse host response, demonstrating its safety and suitability for skin-related applications. Collectively, these findings establish Skin ECM as a bioactive and biocompatible matrix that effectively recapitulates essential features of the native dermal microenvironment. This material offers a scalable and versatile platform for modeling cutaneous regeneration, advancing fibroblast-based assays, and supporting future development of ECM-based solutions in regenerative and cosmetic dermatology.

## Materials and Methods

### Protein Sample Preparation and Liquid Chromatography with Tandem Mass Spectrometry (LC-MS/MS)

To perform proteomic analysis, Skin ECM (#Regenix Skin, #SKdE-1G, Cellartgen, Republic of Korea) and recombinant human collagen III (rhCollagen; Bloomage Biotech, China) were digested using a filter aided sample preparation (FASP) protein digest kit (Abcam, UK). Initially, protein reduction was carried out by treatment with 10 mM DL-dithiothreitol (DTT; Sigma-Aldrich, USA) in a 50 mM ammonium bicarbonate solution at 60°C for 30 min, followed by alkylation with 55 mM iodoacetamide (IAA) in urea solution for 30 min at room temperature while being protected from light. Digestion was performed using mass spectrometry grade trypsin (Promega, USA) at 37°C overnight. After digestion, the supernatants were desalted using Pierce peptide desalting spin columns (Thermo Fisher Scientific, USA). The peptides were dried for 6 h in a centrifugal evaporator (CVE-3000, Eyela, Japan) and then dissolved in a solution of 2% (v/v) acetonitrile (Thermo Fisher Scientific) and 0.1% (v/v) formic acid (TCI, Japan). Peptide concentrations were determined using a Pierce Quantitative colorimetric peptide assay (Thermo Fisher Scientific), and 2 μg of peptides were loaded for LC-MS/MS. LC-MS/MS was performed using the UltiMate 3000 RSLCnano system coupled with the Q-Exactive Orbitrap HF-X mass spectrometer (Thermo Fisher Scientific) using the same materials and settings previously described [17, 18].

### Proteomic Data Analysis

After LC-MS/MS, raw data were processed using MaxQuant (Max Planck Institute of Biochemistry, Germany)(version 2.6.7), with search against the UniProt database of *Sus scrofa* for Skin ECM and *Homo sapiens* for rhCollagen (all released 2025.04) [17]. Protein quantification was based on the label-free quantification (LFQ) with match-between-runs feature. Trypsin was selected as the digesting enzyme, and N-terminal acetylation and methionine oxidation were considered variable modifications in terms of post-translational modifications. Additionally, carbamidomethyl modification was set as a fixed modification. The false discovery rate (FDR) of 0.01 was applied at both the peptide and protein levels. The intensity-based absolute quantification (iBAQ) method was employed for protein quantification. The comparison between different samples was performed using relative iBAQ (riBAQ), which was obtained by dividing the iBAQ value of each protein by the sum of all iBAQ values in each sample.

The proteome of human skin tissue (suprapubic) was derived from the data of a quantitative proteome map to analyze the total and matrisome protein components [18]. The classification of matrisome proteins was based on the MIT Matrisome Project database [19]. Skin-expressed and skin-elevated proteins were searched at the Human Protein Atlas [20]. Gene ontology enrichment analysis was conducted by PANTHER overrepresentation test. The terms with FDR < 0.05 were selected and summarized with REVIGO (http://revigo.irb.hr) [21]. The Voronoi diagram of reactome pathways was created in the Reactome web site (https://reactome.org) [22]. The network of protein–protein interaction (PPI) was generated and visualized using STRING and Cytoscape software, respectively [23, 24]. To illustrate the associations between proteins and their biological functions, a chord plot was created by SRplot [25].

### Cell Culture

Human neonatal dermal fibroblasts (Thermo Fisher Scientific) under passage 7 were cultured in high glucose Dulbecco’s Modified Eagle Medium (DMEM) containing 10% (v/v) fetal bovine serum (FBS) and 1% (v/v) penicillin-streptomycin (P/S) (all from Thermo Fisher Scientific) at 37°C and 5% CO_2_. The cells were trypsinized and seeded at a density of 2,500 cells/cm^2^. In the media supplement experiment, the concentration of FBS in the culture media was adjusted according to the experiment from 0% (serum-free) to 2% (low serum), to clarify the effect of Skin ECM.

### Surface Coating Experiments

For surface coating, lyophilized Skin ECM was solubilized at 10 mg/ml using 2 mg/ml pepsin (Sigma-Aldrich) in 0.01 M hydrochloric acid (HCl; Sigma-Aldrich) at room temperature with stirring. Thereafter, the Skin ECM solution was diluted at 0.01, 0.05, or 0.1 mg/ml in 0.02 M acetic acid (Sigma-Aldrich) and coated on the tissue culture-treated plates for 2 h at 37°C. After washing with phosphate-buffered saline (PBS; Sigma-Aldrich), fibroblasts were seeded on the coated plates. Collagen type I from rat tail (Corning, USA) diluted at 0.05 mg/ml in 0.02 M acetic acid and gelatin (Sigma-Aldrich) dissolved at 1 mg/ml in triple deionized water (TDW) were used as other coating materials for comparison with Skin ECM.

### Measurement of Mitochondrial Activity

The mitochondrial activity of cultured fibroblasts was assessed using 3-(4,5-dimethylthiazol-2-yl)-2,5-diphenyltetrazolium bromide (MTT; Sigma-Aldrich). The cells seeded on 96-well plates were incubated with MTT solution at a concentration of 1 mg/ml for 3-4 h at 37°C. Then, intracellular formazan crystals were dissolved with dimethyl sulfoxide (DMSO; Wako, Japan). The absorbance was measured at 570 nm using a microplate reader (Tecan, Switzerland).

### Cell Viability Test

To examine cell viability, the cultured fibroblasts were stained with Live/Dead viability/cytotoxicity kit (Thermo Fisher Scientific), following the manufacturer’s instructions. The fluorescent images were obtained using a fluorescence microscope (IX73, Olympus, Japan) and quantified with ImageJ software (National Institutes of Health, USA). Cell viability was calculated as the percentage of live cells in the total cells.

### Media Supplement Experiments

For media supplement, Skin ECM solubilized with pepsin was prepared as a pre-gel solution by mixing with 10% (v/v) 10× PBS and 0.5 M sodium hydroxide (NaOH; Sigma-Aldrich) for neutralization. The 5 mg/ml Skin ECM pre-gel solution was added to culture media at final concentrations of 0.01 or 0.05 mg/ml in the transforming growth factor beta 1 (TGF-β1; Peprotech, USA) treatment experiment. Meanwhile, Skin ECM pre-gel solution was added after lyophilization at 1 mg/ml for wound healing assay and pro-collagen secretion measurement.

For details, in the TGF-β1 treatment experiment, the cultured cells were serum-starved for 24 h and then treated with 10 ng/ml TGF-β1 with or without Skin ECM (0.01 or 0.05 mg/ml) for 3 days in serum-free culture media. For the wound healing assay, a linear scratch was generated on the confluent cell monolayer using a 1000p pipette tip. After washing with PBS, the cells were treated with Skin ECM (1 mg/ml) or rhCollagen (1 mg/ml) in serum-free culture media. The wound healing rate was quantified based on brightfield images using ImageJ software. To measure the secreted pro-collagen I α1, the cultured cells were treated with Skin ECM (1 mg/ml) or rhCollagen (1 mg/ml) for 3 days in 2% low serum culture media.

### Measurement of Human Pro-Collagen I α1

The culture media in which fibroblasts were cultured for the last day were collected after 3 days of supplement for enzyme-linked immunosorbent assay (ELISA). The secreted pro-collagen type I was quantified with the human pro-collagen I α1 DuoSet ELISA kit (R&D Systems, USA) according to the manufacturer’s protocols.

### Quantitative Reverse Transcription Polymerase Chain Reaction (qRT-PCR)

For qRT-PCR analysis, RNA was extracted from cultured fibroblasts using the AccuPrep Universal RNA extraction kit (Bioneer, Republic of Korea). cDNA was synthesized from the extracted RNA using the AccuPower CycleScript RT PreMix (Bioneer). The resulting cDNA was amplified using SYBR Green Fast qPCR Mix (ABclonal, USA) on a StepOnePlus Real-time PCR system (Thermo Fisher Scientific). Relative expressions were calculated with the comparative C_T_ method. Specific primers were used to compare expression of the following genes (all primers from Bioneer): human elastin (*ELN*) (Forward: 5’-GGTATCCCATCAAGGCCCC-3’, Reverse: 5’-TTTCCCTGTGGTGTAGGGCA-3’), human alpha-smooth muscle actin (*ACTA2*) (Forward: 5’-GTGAAG AAGAGGACAGCACTG-3’, Reverse: 5’-CCCATTCCCACCATCACC-3’), human collagen type I alpha 1 chain (*COL1A1*) (Forward: 5’-GACGAAGACATCCCACCAATCA-3’, Reverse: 5’-GGACTCGTCACAGATCACGTC-3’), and human interleukin-6 (*IL6*) (Forward: 5’-AAAGAGGCACTGGCAGAAAA-3’, Reverse: 5’-GAGGTG CCCATGCTACATTT-3’). Human glyceraldehyde 3-phosphate dehydrogenase (*GAPDH*) (Forward: 5’-TGC ACCACCAACTGCTTAGC-3’, Reverse: 5’-GGCATGGACTGTGGTCATGAG-3’) was used as a housekeeping gene.

### *In Vitro* and *In Vivo* Biocompatibility Evaluation of Skin ECM

Skin ECM pre-gel solution was used at a concentration of 5 mg/ml for evaluation of biocompatibility both *in vitro* and *in vivo*. For *in vitro* experiment, RAW 264.7 murine macrophages were cultured with Skin ECM hydrogel for 24 h in a transwell system (Corning). Cells in the bottom chamber were cultured in high glucose DMEM containing 10% (v/v) FBS and 1% (v/v) P/S, and 50 μl of Skin ECM hydrogel was added to the upper insert (0.4 μm pore). Lipopolysaccharide (LPS; Sigma-Aldrich) was treated as a positive control at 1 μg/ml concentration. After 24 h, the culture media were collected to quantify tumor necrosis factor alpha (TNF-α) and interleukin-6 (IL-6) secretion using the corresponding ELISA kits (R&D Systems), following the manufacturer’s protocols.

*In vivo* biocompatibility experiment was approved by the Institutional Animal Care and Use Committee (IACUC) of Yonsei University (IACUC-A-202504-2032-01). During the experiment, mice were maintained under a controlled environment (21 ± 2°C, 50 ± 10% humidity, ventilation of 10–15/h, 150–300 Lux light intensity, and noise < 60 dB). After anesthesia with ketamine (100 mg/kg; Yuhan, Republic of Korea) and xylazine (20 mg/kg; Bayer Korea, Republic of Korea), 200 μl of Skin ECM pre-gel solution was subcutaneously injected into the dorsal region of the mouse. As a control group, mice were injected with the same volume of PBS. At 1 and 14 days post-injection, skin and major organs (heart, lung, liver, kidney, and spleen) were harvested and fixed in 10% neutral buffered formalin (Sigma-Aldrich). The fixed tissue samples were embedded in paraffin and sectioned at 7 μm thickness for hematoxylin and eosin (H&E) and toluidine blue (TB) staining. The stained slides were visualized with a slide scanner (VS120-S5-W, Olympus). The body and five major organs of the mice were weighed before fixation.

### Statistical Analysis

Quantitative data are presented as mean ± standard deviation (SD), with the number of biological replicates (*n*) specified in the corresponding figure legends. Statistical analyses were performed with GraphPad Prism 10 (GraphPad Software, USA). Statistical significance was determined using unpaired two-tailed *t*-test or one-way analysis of variance (ANOVA) with Tukey's multiple comparisons test, as described in the corresponding figure legends.

## Results

### Skin ECM Proteomics Identifies Key Bioactive Molecules Involved in Dermal Repair

**Fig. 1:**
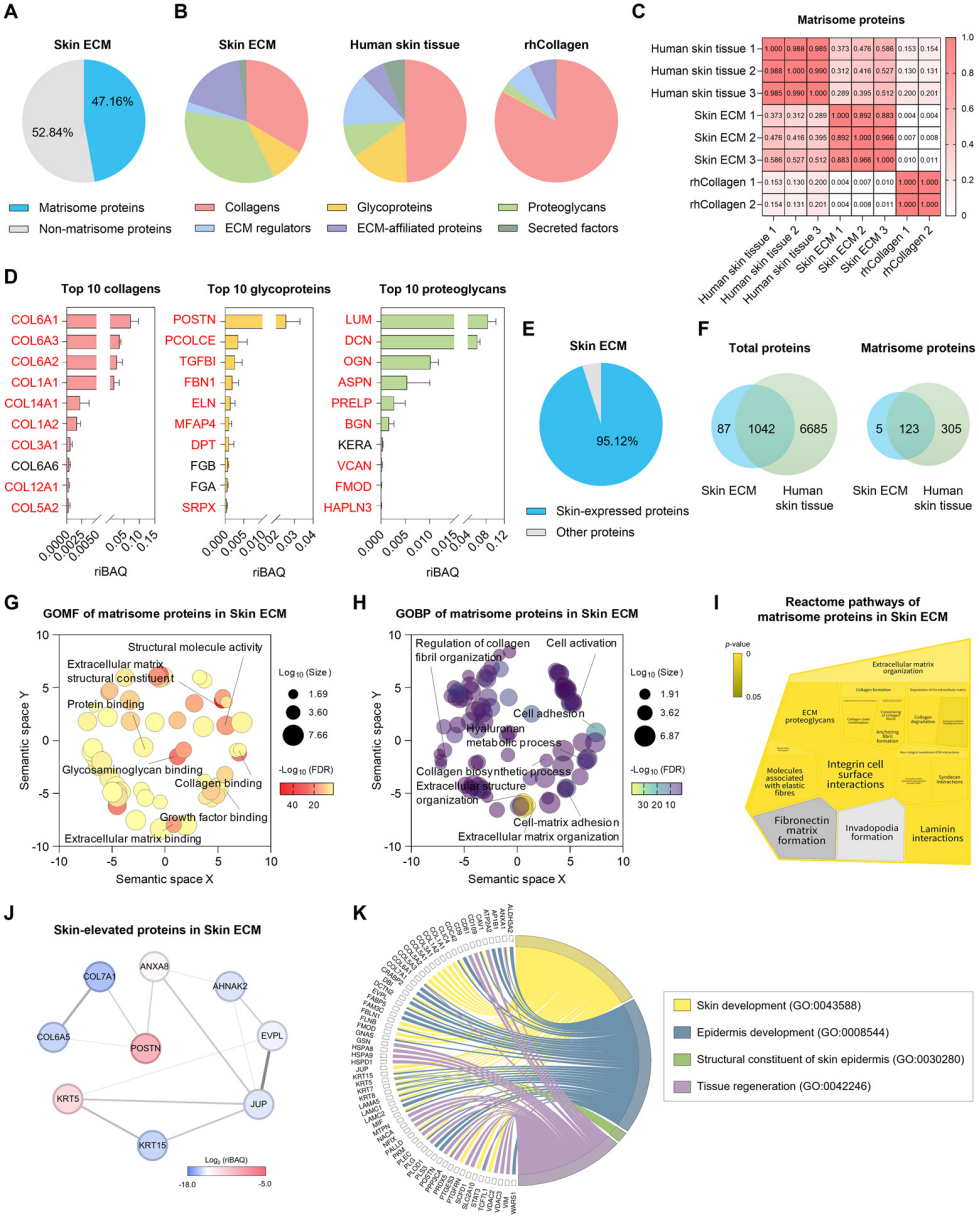
Proteomic characterization of Skin ECM reveals tissue-specific composition and enrichment of dermal matrisome proteins. (**A**) Pie chart showing the proportion of ECM proteins (based on the MatrisomeDB classification) in Skin ECM. (**B**) Distribution of matrisome subcategories within Skin ECM, human skin tissue, and recombinant human collagen III (rhCollagen), including core matrisome components (collagens, glycoproteins, proteoglycans) and matrisome-associated proteins (ECM regulators, ECM-affiliated proteins, secreted factors). (**C**) Correlation matrix with Pearson correlation coefficients between matrisome protein expression in human skin tissue, Skin ECM, and rhCollagen. (**D**) Top 10 most abundant proteins in the core matrisome categories of Skin ECM, including collagens, glycoproteins, and proteoglycans. Proteins shown in red indicate to be expressed in the native human skin tissue. (**E**) Pie chart showing the proportion of Skin ECM proteins that are annotated as skin-expressed proteins (95.12% by total abundance), based on the Human Protein Atlas. (**F**) Venn diagrams showing protein overlap between Skin ECM and human skin proteomes at the level of total proteins (left) and matrisome proteins (right). Scatterplots of (**G**) gene ontology molecular function (GOMF) and (**H**) gene ontology biological process (GOBP) terms significantly enriched in matrisome proteins of Skin ECM. The terms were positioned based on semantic similarity using REVIGO. The bubble size represents the log-transformed ‘Size’, which corresponds to the number of annotations for each term in the European Bioinformatics Institute Gene Ontology Annotation (EBI GOA) database. (**I**) A Voronoi diagram illustrating the Reactome pathway analysis results of matrisome proteins in Skin ECM. Color intensity indicates the *p*-value from the statistical test. Pathways shown in dark grey are not significantly overrepresented, while those with no associated proteins are depicted in light grey. (**J**) Protein–protein interaction (PPI) network of skin-elevated proteins in Skin ECM. Node color represents relative abundance. (**K**) A chord plot showing relationships between GOBP terms (skin development, epidermis development, structural constituent of skin epidermis, and tissue regeneration) and associated proteins in Skin ECM.

To characterize the protein composition of Skin ECM, mass spectrometry-based proteomic analysis was performed and compared with rhCollagen and native human skin tissue (reference dataset from human proteome map) [18]. Classification using the Matrisome Project database [19] revealed that 47.16% of the total proteins identified in Skin ECM were matrisome proteins (Fig. 1A). Composition analysis indicated that Skin ECM contained all six ECM categories in proportions similar to native skin, while rhCollagen was composed almost exclusively of collagens, lacking broader matrisome diversity (Fig. 1B). Pearson correlation analysis confirmed that Skin ECM exhibited a higher similarity in protein composition to human skin tissue than rhCollagen (Fig. 1C).

Among the top 10 most abundant core matrisome proteins in Skin ECM, a majority belonged to the collagen family, including *COL1A1*, collagen type VI alpha 1 and alpha 2 chains (COL6A1, COL6A2), and type XIV alpha 1 chain (COL14A1). While *COL1A1* and COL6A family members are abundantly expressed structural collagens in the dermis, COL14A1 is a fibril-associated collagen that modulates collagen fiber organization and is expressed at lower levels in adult skin [18, 26] (Fig. 1D, left panel). Skin ECM also contained a diverse set of glycoproteins associated with dermal architecture and fibroblast function. These included periostin (POSTN), a matricellular protein enriched at sites of matrix remodeling [27], and elastin (ELN), critical for skin elasticity [28]. Other glycoproteins such as procollagen C-endopeptidase enhancer (PCOLCE) were also abundant, suggesting the presence of regulatory components that facilitate collagen fibrillogenesis and matrix organization [29] (Fig. 1D, middle panel). In the proteoglycan category, highly abundant proteins included lumican (LUM) decorin (DCN), and biglycan (BGN) — all of which play critical roles in collagen fibrillogenesis and matrix stability [30-32]. Additional identified proteoglycans such as versican (VCAN) and fibromodulin (FMOD) are known contributors to dermal ECM structure and skin homeostasis [33-35] (Fig. 1D, right panel). Notably, many of these proteoglycans are also upregulated during wound healing, underscoring the functional relevance of Skin ECM for supporting dermal tissue repair.

Further analysis showed that 95.12% of the proteins identified in Skin ECM are encoded by genes known to be expressed in human skin, indicating strong tissue specificity (Fig. 1E). In total, 1,042 out of 1,129 proteins overlapped with the human skin proteome, while 123 of the 128 matrisome proteins in Skin ECM were also found in native skin tissue (Fig. 1F). Together, these findings demonstrate that Skin ECM retains a broad and skin-relevant repertoire of collagens, glycoproteins, and proteoglycans, including many proteins that are highly expressed in the dermis. This compositional fidelity supports the ability of Skin ECM to recapitulate key structural and signaling features of the native dermal ECM.

Gene ontology molecular function (GOMF) enrichment of Skin ECM matrisome proteins revealed significant associations with collagen binding, ECM structural constituent, and structural molecule activity (Fig. 1G). Gene ontology biological process (GOBP) analysis further identified enrichment in processes related to hyaluronan metabolic process, cell activation and regulation of collagen fibril organization (Fig. 1H). Reactome pathway mapping indicated that many Skin ECM proteins are associated with ECM organization pathways, including integrin cell surface interactions, laminin interactions, and molecules associated with elastic fibers (Fig. 1I). PPI analysis of skin-elevated proteins revealed a tightly connected network of structural and signaling proteins, with abundant proteins such as POSTN, collagen type VII alpha 1 chain (COL7A1), and collagen type VI alpha 5 chain (COL6A5) acting as central nodes (Fig. 1J). Finally, a chord diagram linking Skin ECM proteins to GOBP terms highlighted the inclusion of numerous proteins involved in skin development, epidermis development, structural constitution of the epidermis, and tissue regeneration (Fig. 1K). Together, these findings demonstrate that Skin ECM preserves the key protein composition, molecular functions, and pathway-level complexity of native dermal ECM, supporting its application as a biologically instructive material for dermal modeling and regenerative therapies.

### Skin ECM Promotes Regenerative and Anti-Inflammatory Responses While Maintaining Proliferative Homeostasis

**Fig. 2:**
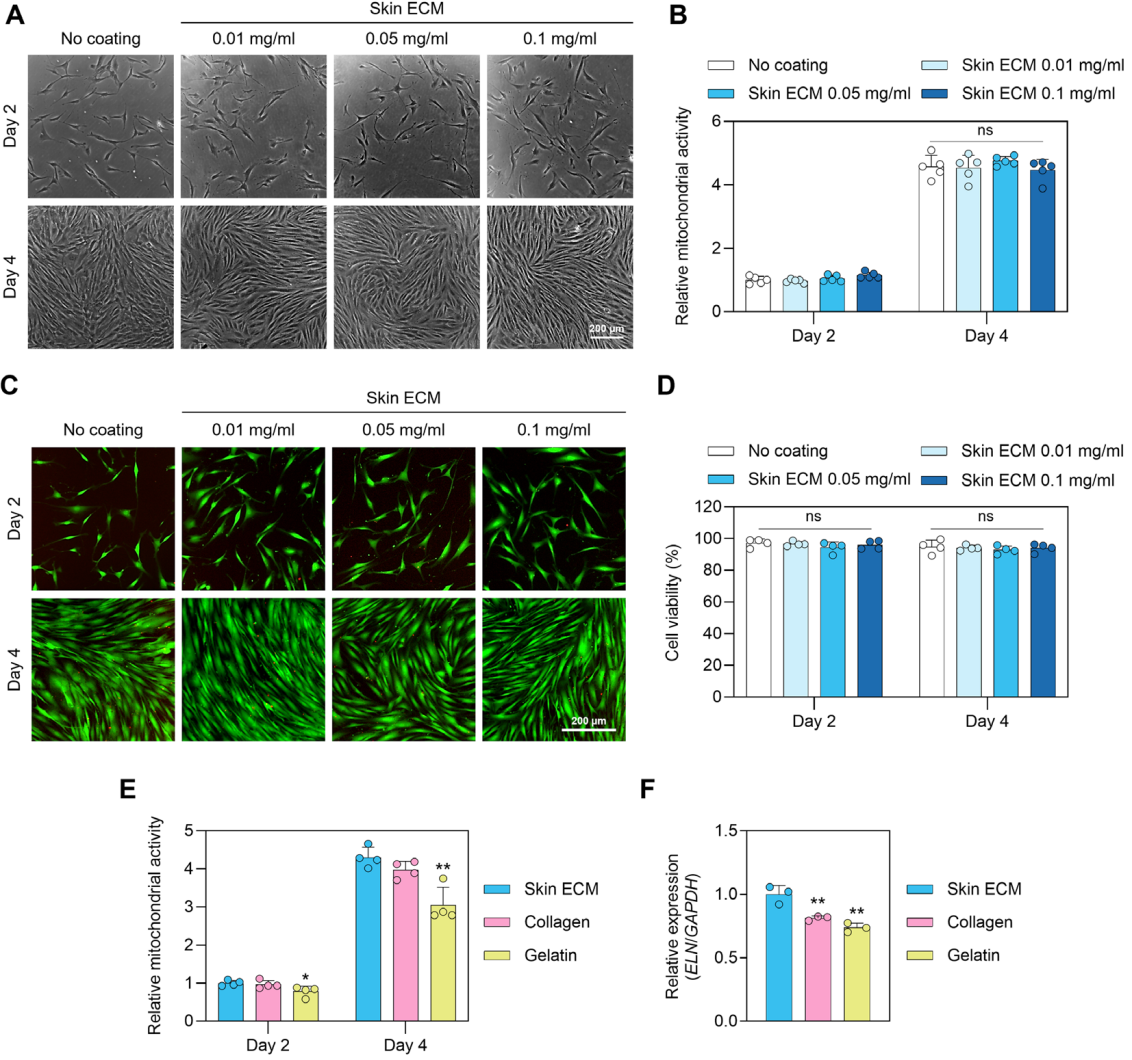
Skin ECM coating supports fibroblast viability and matrix gene expression without inducing hyperproliferation. (**A**) Phase-contrast images of human dermal fibroblasts cultured on tissue culture surfaces coated with increasing concentrations of Skin ECM (0, 0.01, 0.05, and 0.1 mg/ml) at day 2 and day 4. Scale bar = 200 μm. (**B**) MTT assay showing mitochondrial activity at day 2 and day 4 (*n* = 5; ns: not significant). (**C**) Representative Live/Dead fluorescence staining images of fibroblasts at each coating concentration. Green: live cells; red: dead cells. Scale bar = 200 μm. (**D**) Quantification of cell viability based on Live/Dead staining (*n* = 4; ns: not significant). (**E**) MTT assay comparing fibroblast viability on Skin ECM (0.05 mg/ml), type I collagen from rat tail (0.05 mg/ml), or gelatin (1 mg/ml) (*n* = 4, *p < 0.05 and **p < 0.01 versus Skin ECM). (**F**) qRT-PCR analysis of elastin (ELN) expression in fibroblasts cultured on Skin ECM (0.1 mg/ml), type I collagen from rat tail (0.05 mg/ml), or gelatin (1 mg/ml) (*n* = 3, **p < 0.01 versus Skin ECM). Data are presented as mean ± SD. Statistical significance was assessed by one-way ANOVA followed by Tukey’s multiple comparisons test.

To assess the cytocompatibility and bioactivity of Skin ECM coatings, dermal fibroblasts were cultured on surfaces coated with varying concentrations of Skin ECM (0.01–0.1 mg/ml), and mitochondrial activity was measured by MTT assay. No significant differences were observed among the concentrations, with comparable levels of metabolic activity maintained across all groups (Fig. 2A and 2B). These results suggest that Skin ECM provides a supportive microenvironment for fibroblast survival without inducing abnormal or excessive proliferation. Live/Dead fluorescence imaging further confirmed the cytocompatibility of Skin ECM coatings. Viable fibroblasts were observed at all concentrations, exhibiting uniform morphology and minimal cytotoxicity (Fig. 2C). Quantitative analysis revealed no significant difference in live cell proportions across coating concentrations (Fig. 2D), indicating the stability and safety of the Skin ECM surface within this range. To compare Skin ECM with widely used ECM substrates, fibroblasts were cultured on surfaces coated with Skin ECM (0.05 mg/ml), type I collagen (0.05 mg/ml), or gelatin (1 mg/ml). MTT analysis revealed that Skin ECM supported fibroblast viability at levels comparable to or greater than those observed with conventional substrates (Fig. 2E).

To further investigate its functional influence on ECM remodeling, gene expression analysis by qRT-PCR was performed. Fibroblasts cultured on Skin ECM (0.1 mg/ml) exhibited significantly elevated expression of elastin compared to collagen- and gelatin-coated groups (Fig. 2F). This suggests that Skin ECM promotes the transcriptional activation of matrix components involved in elastic fiber formation, reinforcing its role in supporting physiologically relevant ECM remodeling. Collectively, these results demonstrate that Skin ECM, when applied as a coating, provides a biologically instructive and homeostatic environment that supports fibroblast viability, preserves cellular morphology, and promotes matrix-associated gene expression without inducing uncontrolled growth.

**Fig. 3:**
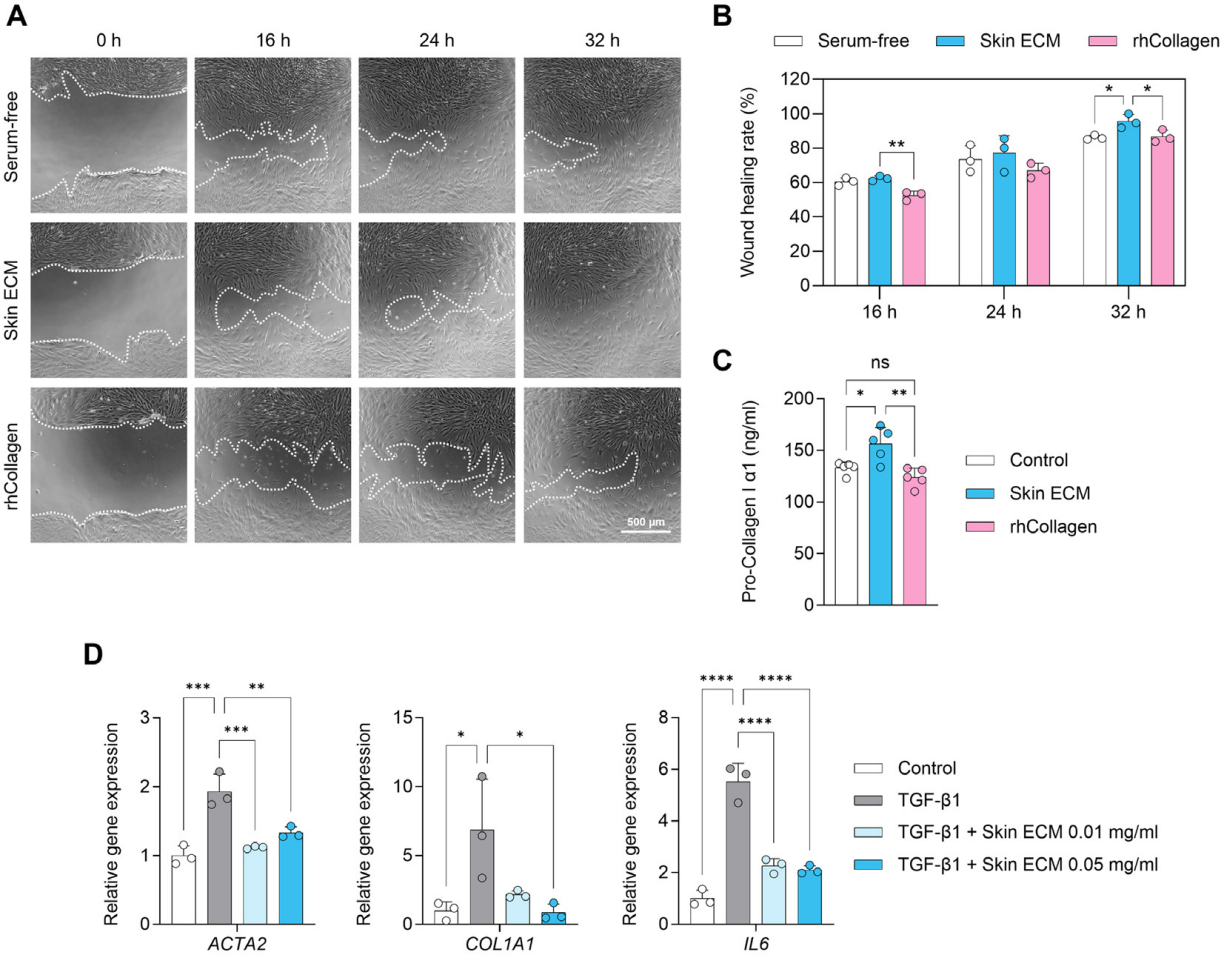
Skin ECM supplement enhances fibroblast migration and suppresses profibrotic and inflammatory gene expression. (**A**) Representative scratch wound healing images of human dermal fibroblasts treated with vehicle control (serum-free), Skin ECM (1 mg/ml), or rhCollagen (1 mg/ml) at 0 to 32 h. Dotted lines delineate wound boundaries. Scale bar = 500 μm. (**B**) Quantification of wound closure at 32 h demonstrates significantly increased fibroblast migration in the Skin ECM group compared to control and rhCollagen-treated groups (*n* = 3, *p < 0.05 and **p < 0.01 versus indicated group). (**C**) ELISA results showing levels of secreted pro-collagen type I in fibroblast cultures treated with vehicle (low serum), Skin ECM (1 mg/ ml), or rhCollagen (1 mg/ml). Skin ECM increased collagen output compared to control (*n* = 5; *p < 0.05 and **p < 0.01; ns: not significant). (**D**) Gene expression analysis of *ACTA2*, *COL1A1*, and *IL6* in fibroblasts stimulated with TGF-β1 (10 ng/ml), with or without co-treatment with Skin ECM (0.01 or 0.05 mg/ml) (*n* = 3, *p < 0.05, **p < 0.01, ***p < 0.001, and ****p < 0.0001 versus indicated group). Data are presented as mean ± SD. Statistical analysis was performed using one-way ANOVA followed by Tukey’s multiple comparisons test.

Next, the biological activity of Skin ECM as a soluble supplement was evaluated in serum-free or low serum conditions using primary human dermal fibroblasts. To assess regenerative function, fibroblast motility was analyzed using an *in vitro* scratch assay. Fibroblasts treated with Skin ECM exhibited significantly accelerated wound closure over 32 h compared to the untreated group, whereas treatment with rhCollagen did not elicit a comparable response (Fig. 3A and 3B). These results suggest that Skin ECM enhances fibroblast-mediated wound repair primarily by promoting migration rather than cell proliferation. Matrix remodeling capacity was further evaluated by quantifying pro-collagen type I secretion. Fibroblasts treated with Skin ECM tended to secrete higher levels of collagen compared to both untreated and rhCollagen-treated groups (Fig. 3C), indicating a potential stimulatory effect on ECM production.

To examine antifibrotic and anti-inflammatory properties, gene expression was analyzed in fibroblasts stimulated with TGF-β1, a known inducer of myofibroblast differentiation [36]. As expected, TGF-β1 stimulation markedly upregulated *ACTA2*, *COL1A1*, and *IL6*, indicative of fibroblast activation and inflammatory signaling. Co-treatment with Skin ECM at concentrations of 0.01 mg/ml and 0.05 mg/ml significantly attenuated the expression of all three genes, restoring them to levels similar to those of untreated fibroblasts (Fig. 3D).

Together, these results demonstrate that Skin ECM, when applied as a soluble supplement, supports fibroblast migration and matrix remodeling while maintaining proliferative homeostasis. Furthermore, Skin ECM suppresses TGF-β1-induced expression of key fibrotic and inflammatory markers, underscoring its dual role as a regenerative and immunomodulatory matrix component suitable for skin repair and therapeutic applications.

### Skin ECM Demonstrates High *In Vivo* Biocompatibility without Adverse Host Response

**Fig. 4:**
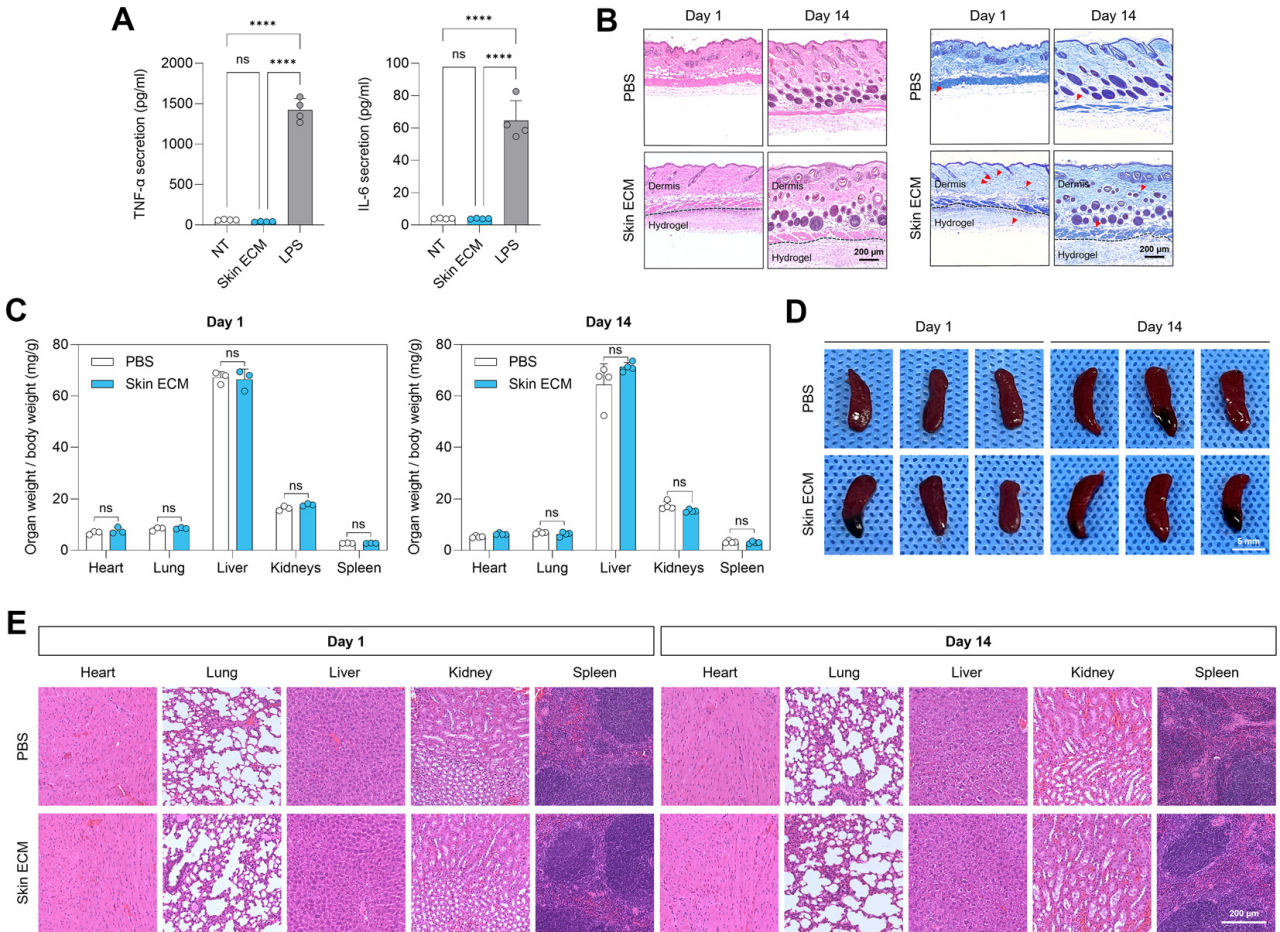
Skin ECM exhibits excellent biocompatibility *in vitro* and *in vivo*. (**A**) ELISA quantification of TNF-α and IL-6 secretion from RAW 264.7 macrophages treated with Skin ECM (5 mg/ml) or LPS (1 μg/ml) stimulation. Skin ECM did not induce pro-inflammatory cytokine release (*n* = 4; ****p < 0.0001, ns: not significant; one-way ANOVA with Tukey’s post hoc test). (**B**) Representative histological images (H&E and TB staining) of skin tissue at the subcutaneous injection site 1 and 14 days after implantation of Skin ECM (5 mg/ml). Dashed lines denote the boundary between the mouse skin layers and hydrogels. Mast cells with purple granules are indicated with red arrowheads in the TB-stained images. Scale bars = 200 μm. (**C**) Quantification of organ-to-body weight ratios (mg/g) for heart, lung, liver, kidney, and spleen at days 1 and 14 following subcutaneous administration of Skin ECM or PBS (*n* = 3–4 per group; ns: not significant; unpaired two-tailed *t*-test). (**D**) Macroscopic spleen images showing no morphological abnormalities or splenomegaly in Skin ECM-treated mice compared to PBS controls. Scale bar = 5 mm. (**E**) H&E staining of major organs (heart, lung, liver, kidney, and spleen) 1 and 14 days postinjection. Scale bar = 200 μm. Data are presented as mean ± SD.

To evaluate the immunomodulatory effects of Skin ECM *in vitro*, RAW 264.7 murine macrophages were co-cultured with Skin ECM (5 mg/ml) and compared with untreated (no treatment) and LPS-treated groups. As expected, LPS stimulation markedly increased the secretion of pro-inflammatory cytokines TNF-α and IL-6. In contrast, macrophages treated with Skin ECM did not exhibit elevated cytokine levels, and levels remained comparable to the untreated control (Fig. 4A). These results indicate that Skin ECM does not provoke an inflammatory response and suggest potential anti-inflammatory or immunomodulatory properties.

The *in vivo* biocompatibility of Skin ECM was assessed through subcutaneous implantation in mice for 1 and 14 days. Histological evaluation of the implantation site revealed minimal immune cell infiltration and no evidence of fibrotic encapsulation. H&E and TB staining of skin tissue confirmed that Skin ECM did not elicit a localized inflammatory or fibrotic response (Fig. 4B).

To assess potential systemic toxicity, major organ weights were recorded and normalized to body weight. No significant differences were observed in heart, lung, liver, kidney, or spleen weights between the Skin ECM and control groups at either time point (Fig. 4C). Macroscopic evaluation of the spleen (Fig. 4D) and histological analysis of all major organs (heart, lung, liver, kidney, and spleen) using H&E staining (Fig. 4E) revealed no pathological changes, confirming the absence of off-target tissue damage or systemic inflammatory burden. Together, these findings demonstrate that Skin ECM is well tolerated *in vivo*, exhibiting high local and systemic biocompatibility without eliciting inflammatory or toxic responses. These properties support its safe application in regenerative and therapeutic settings.

## Discussion

Decellularized ECMs derived from native tissues offer complex biochemical environments that are difficult to replicate with single-component substrates. In this study, we assessed the functional properties of Skin ECM, a decellularized dermal matrix formulated for use as both a soluble supplement and a surface coating, and demonstrated its ability to recreate key microenvironmental features of native skin ECM *in vitro* and *in vivo*.

Proteomic analysis confirmed that Skin ECM retains a skin-specific matrisome profile enriched in structural and regulatory components, including fibrillar collagens, glycoproteins, and proteoglycans known to regulate fibroblast behavior and matrix assembly. The high degree of overlap with the human skin proteome highlights its compositional fidelity, which is crucial for maintaining cell–matrix signaling relevant to wound healing, remodeling, and tissue homeostasis. Notably, proteins such as fibronectin, periostin, elastin, lumican, decorin, and biglycan—each implicated in collagen organization, fibroblast activation, and skin elasticity—were preserved, supporting Skin ECM’s biological functionality.

Unlike recombinant collagen or gelatin, Skin ECM promoted fibroblast migration while maintaining a non-proliferative, homeostatic state. This distinction is especially important in regenerative modeling, where overstimulation of fibroblast growth may compromise physiological relevance or exaggerate fibrotic responses. Enhanced motility observed in scratch assays likely reflects integrin-mediated interactions with retained matrix ligands, which coordinate directional migration during tissue repair.

The increased production of collagen type I in Skin ECM-treated fibroblasts suggests that Skin ECM contributes to ECM remodeling. Of particular significance was its ability to suppress TGF–β1–induced expression of *ACTA2*, *COL1A1*, and *IL6*, indicating an inhibitory effect on fibroblast-to-myofibroblast transition and pro-inflammatory signaling. This dual regulatory capacity may arise from matrix-bound factors or structural components that interfere with canonical profibrotic pathways, an effect consistent with known functions of decorin and fibromodulin in modulating TGF-β activity [37, 38].

Importantly, *in vivo* implantation studies showed that Skin ECM was well tolerated, with no detectable immune response or systemic toxicity. This biocompatibility, together with its demonstrated biological activity, underscores its suitability for translational applications. Unlike bulk dermal grafts or injectable hydrogels, the use of Skin ECM in coating or supplement form offers practical advantages for *in vitro* systems, including ease of integration into standardized workflows and compatibility with high-throughput formats.

Nevertheless, this study has limitations. Proteomic data does not capture post-translational modifications or the activity of retained growth factors, both of which could influence fibroblast responses. All experiments were performed in two-dimensional (2D) culture systems, which lack the architectural and mechanical complexity of native skin. Future studies should explore the integration of Skin ECM into 3D tissue constructs and assess its performance in co-culture systems with keratinocytes, endothelial cells, or immune cells. Furthermore, the signaling pathways by which Skin ECM modulates fibroblast activation remain to be defined and may involve non-canonical regulators beyond TGF-β/Smad signaling [39].

In summary, Skin ECM functions as a biologically active, tissue-specific, and immunologically compatible ECM material that supports fibroblast viability, motility, and matrix remodeling while suppressing pathological activation. Its accessible format, physiological relevance, and safety profile make it a promising tool for regenerative studies, fibrosis modeling, and the development of next-generation dermal therapeutics and cosmetic biomaterials.

Considering the translational applications, further investigation is required for the standardized and large-scale production of Skin ECM. As decellularized ECM is inherently derived from biological sources, it is subject to batch-to-batch variability. Regarding the source organs for decellularization, donor characteristics (*e.g.*, age and sex) and tissue region influence the variability in the resulting decellularized ECM; therefore, securing source organs with consistent profiles can minimize such variations [40-42]. In addition, optimizing the decellularization protocols can address batch-to-batch variability in terms of residual components and hydrogel stability [43, 44]. Recent advancements in decellularization processes—including the development of automated systems and implementation of in-process monitoring—have also contributed to reducing batch-to-batch variations of decellularized ECM [45, 46]. As part of rigorous quality control, proteomic analysis can ensure the preservation of essential ECM components and tissue-specific proteins [41, 42]. Along with product reproducibility, a more simplified and economically optimized manufacturing process can also improve the feasibility of the scalable production of Skin ECM. From a functional perspective of *in vivo* wound healing efficiency, decellularized skin has been reported to accelerate wound closure, accompanied by collagen deposition and anti-inflammation [47-49]. Our study validated the capacity of Skin ECM to drive ECM remodeling and suppress pro-inflammatory signaling *in vitro*, suggesting its potential for the *in vivo* wound healing process.
